# The effect of four-phasic versus three-phasic contrast media injection protocols on extravasation rate in coronary CT angiography: a randomized controlled trial

**DOI:** 10.1007/s00330-017-4866-0

**Published:** 2017-05-24

**Authors:** Júlia Karády, Alexisz Panajotu, Márton Kolossváry, Bálint Szilveszter, Ádám L. Jermendy, Andrea Bartykowszki, Mihály Károlyi, Csilla Celeng, Béla Merkely, Pál Maurovich-Horvat

**Affiliations:** MTA-SE Cardiovascular Imaging Research Group, Heart and Vascular Center, Semmelweis University, 68 Varosmajor Street, 1122 Budapest, Hungary

**Keywords:** Computed tomography angiography, Contrast media, Contrast media extravasation, Multidetector-row computed tomography, Coronary heart disease

## Abstract

**Objectives:**

Contrast media (CM) extravasation is a well-known complication of CT angiography (CTA). Our prospective randomized control study aimed to assess whether a four-phasic CM administration protocol reduces the risk of extravasation compared to the routinely used three-phasic protocol in coronary CTA.

**Methods:**

Patients referred to coronary CTA due to suspected coronary artery disease were included in the study. All patients received 400 mg/ml iomeprol CM injected with dual-syringe automated injector. Patients were randomized into a three-phasic injection-protocol group, with a CM bolus of 85 ml followed by 40 ml of 75%:25% saline/CM mixture and 30 ml saline chaser bolus; and a four-phasic injection-protocol group, with a saline pacer bolus of 10 ml injected at a lower flow rate before the three-phasic protocol.

**Results:**

2,445 consecutive patients were enrolled (mean age 60.6 ± 12.1 years; females 43.6%). Overall rate of extravasation was 0.9% (23/2,445): 1.4% (17/1,229) in the three-phasic group and 0.5% (6/1,216) in the four-phasic group (p = 0.034).

**Conclusions:**

Four-phasic CM administration protocol is easy to implement in the clinical routine at no extra cost. The extravasation rate is reduced by 65% with the application of the four-phasic protocol compared to the three-phasic protocol in coronary CTA.

***Key Points*:**

*• Four-phasic CM injection-protocol reduces extravasation rate by 65% compared to three-phasic.*

*• The saline pacer bolus substantially reduces the risk of CM extravasation.*

*• The implementation of four-phasic injection-protocol is at no cost.*

## Introduction

To achieve robust diagnostic performance in coronary computed tomography angiography (CTA) adequate intraluminal iodinated contrast media (CM) concentration is required. Therefore, high flow rate injection, high concentration and relatively large volume of CM is used in daily practice. However, the highly viscous iodinated CM and the high injection flow rate increase the risk of vessel wall injury resulting in CM extravasation.

Contrast media extravasation is a well-known complication of CTA, with an incidence rate of 0.3–1.3% [1–6]. In case of CM extravasation, image quality is deteriorated due to insufficient intraluminal attenuation [7, 8], leading to an increased number of repeated CTA examinations, which results in extra radiation doses, additional CM load and increased costs. Extravasation usually resolves without any serious complications; however, in some instances it can lead to severe injuries [9]. CM has toxic effects on perivascular tissues that may trigger acute and chronic local inflammatory response, tissue necrosis or compartment syndrome [4, 5, 10, 11]. It has been shown that female gender, elderly age, history of chemo- or radiotherapy, low muscle volume and peripheral locations other than the cubital region as injection site increase the risk of CM extravasation [2, 3, 12].

A three-phasic CM injection-protocol is widely used to achieve optimal attenuation during coronary CTA, which results in high contrast enhancement in the left side of the heart and in a lower enhancement in the right [13, 14]. The traditional three-phasic injection-protocol starts with a high flow rate CM injection (>5 ml/s), continues with a mixture of CM and saline, and finishes with a saline chaser bolus. We hypothesized that the relatively large quantity of high viscosity CM could place an increased strain on the vein’s wall, which increases the risk of extravasation. Extending the three-phasic injection-protocol with an initial slower saline flux of pacer bolus right before CM administration may open the possibly collapsed vein lumen with less stress on the vessel wall, thus when the contrast material enters the lumen with a higher flow rate, the already pre-dilated lumen is less likely to rupture. Therefore, we sought to evaluate the effect of a four-phasic contrast injection-protocol on the CM extravasation rate in coronary CTA in a randomized controlled clinical trial.

## Materials and methods

### Study design

In this prospective, single-centre, single-blinded, randomized, controlled clinical trial we compared two contrast media injection-protocols in patients who were referred for coronary CTA examination. We randomized patients into two groups: (1) a three-phasic injection-protocol group and (2) a four-phasic injection-protocol group. To achieve randomization, we alternated the use of three-phasic and four-phasic CM administration protocols on a weekly basis: on even weeks, we applied the three-phasic protocol, and on odd weeks we used the four-phasic protocol. The primary endpoint was the occurrence of CM extravasation. CM extravasation was defined as (1) presence of pain and local swelling close to the cannula insertion site occurring after the initiation of CM injection, and (2) absence of CM or minimal CM attenuation in cardiac chambers on the CTA images. Our institutional review board approved the study and informed consent was waived.

### Study population and CTA protocol

We included consecutive patients who were referred for coronary CTA from January 2014 to August 2015. Exclusion criteria were contraindications to iodinated CM, age under 18 years and the presence of a cannula that was not inserted by our radiographers.

We performed all coronary CTA examinations with a 256-slice multi-detector row CT scanner (Brilliance iCT 256; Philips Healthcare, Best, The Netherlands). Contrast-enhanced image acquisition was performed in inspiration during a single breath-hold in a cranio-caudal direction. The following imaging parameters were used: slice collimation of 128 mm × 0.625 mm, rotation time of 270 ms, tube voltage 100–120 kV and tube current 150–300 mAs depending on the patient’s weight. The majority of scans (99.8%) were acquired by using prospective ECG triggering at 78% phase of the cardiac cycle with 3% padding. Bolus tracking was used with a region of interest (ROI) placed in the left atrium. Images were reconstructed with a slice thickness of 0.8 mm and 0.4 mm increment.

### Contrast media injection-protocol

All patients received the same type of cannula (B. Braun Medical Inc., Melsungen, Germany). All cannulas were inserted by certified radiographers. The preferred location of vein puncture was the right antecubital region. Other distal venous access locations were used if no suitable vein was found in the antecubital region. We registered venipuncture characteristics, such as the side and location of venous access, the size of the inserted cannula and the number of insertion attempts. In all patients the injection site was tested with a 20-ml saline bolus. All patients received a 400 mg/ml concentration iomeprol (Iomeron 400, Bracco Spa, Milan, Italy) CM injected with dual-syringe automated mechanical injector. CM was pre-heated to 37 °C. In patients having more than 80 kg of bodyweight we used a 5.5 ml/s injection rate and 95 ml CM and 120 kV tube voltage. In patients less than 80 kg in bodyweight we used an injection rate of 4.5 ml/s, 80 ml CM and 100 kV tube voltage.

**Fig. 1:**
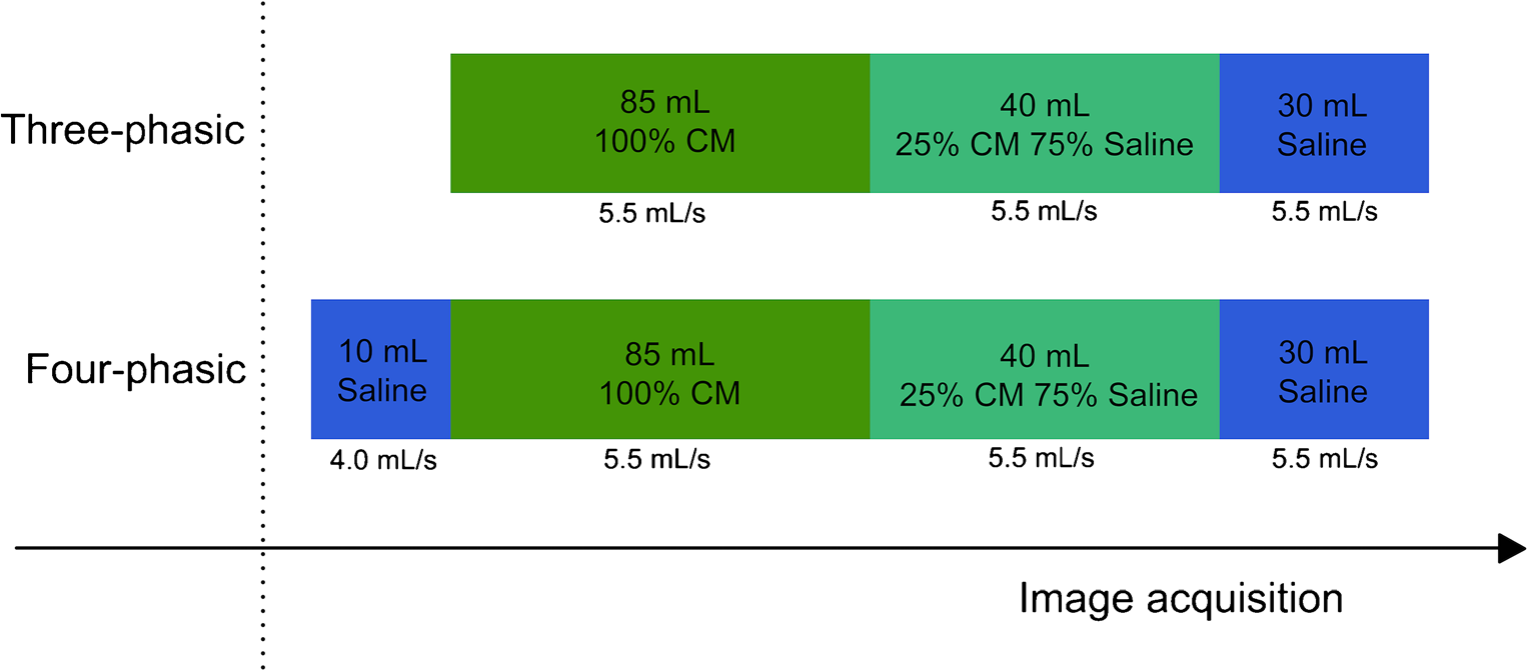
Schematic representation of the three-phasic and the four-phasic contrast media (CM) injection-protocols. The three-phasic protocol starts with an undiluted CM bolus, followed by a 75%:25% saline and CM mixture and ends with a 30-ml chaser saline bolus. The four-phasic protocol starts with a 10-ml saline pacer bolus, administered at a 1.5 ml/s slower flow rate than the CM bolus, and continues with the three-phasic protocol. The injection rate settings are dependent on the body weight of the patient and on the tube voltage settings

In the three-phasic protocol group the injection started with the CM bolus, followed by 40 ml of 75%:25% saline-CM mixture, and finished with 30 ml of chaser saline bolus. With the four-phasic protocol the injection started with the saline pacer bolus of 10 ml, administered with 1.5 ml/s lower flow rate than the CM bolus; specifically, a saline pacer bolus flow rate of 4.0 ml/s if the injection rate of CM was 5.5 ml/s and 3 ml/s if the CM flow rate was 4.5 ml/s and continued with the steps of the three-phasic protocol (Fig. 1).

### Statistical analysis

Continuous variables are reported as mean ± standard deviation. Based on our relatively large sample size the central limit theorem allows the use of parametric tests, therefore we compared the continuous variables using Student’s t-test. To evaluate the differences between categorical variables we used Fisher’s exact test. Two-sided P values below 0.05 were considered statistically significant. For risk estimation, we calculated the odds ratio (OR) with 95% confidence intervals (CIs). All statistical data analysis was performed with IBM SPSS (IBM Corp: version 23, Armonk, NY, USA).

## Results

In total, 2,445 consecutive patients with suspected coronary artery disease were enrolled between 2014 January and 2015 August. The mean age was 60.6 ± 12.1 years and there were less female patients than males (females 43.6%). The clinical characteristics of included patients are summarized in Table 1.

**Table 1:** Clinical characteristics of the patients

	n = 2445
Age (y)	60.6 ± 12.1
Female (%)	1,065 (43.6)
Height (cm)	171.2 ± 10.1
Weight (kg)	84.0 ± 17.2
BMI (kg/m^2^)	28.5 ± 4.8
Hypertension (%)	1,605 (65.6)
Diabetes (%)	393 (16.1)
Dyslipidaemia (%)	1,142 (46.7)
AMI (%)	185 (7.6)
PAD (%)	236 (9.7)
Stroke/TIA (%)	135 (5.5)
Current smoking (%)	990 (40.5)
Total DLP (mGy*cm)	356.8 ± 142.0
Effective dose (mSv)	5.0 ± 2.0
Contrast material (ml)	91.2 ± 7.3

*AMI* acute myocardial infarction, *BMI* body mass index, *PAD* peripheral artery disease, *TIA* transient ischaemic attack, *DLP* dose-length product

**Fig. 2:**
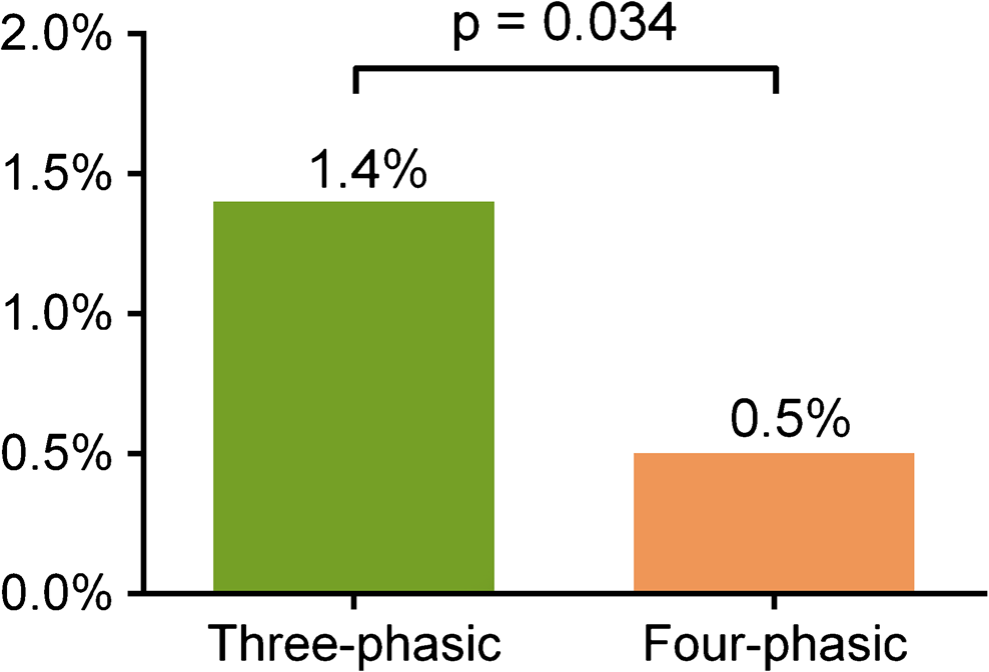
The contrast media extravasation rate in the three-phasic group was 1.4% (17/1,229), whereas in the four-phasic contrast media injection-protocol group the extravasation rate was 0.5% (6/1,216), p = 0.034

Out of the 2,445 patients, 1,229 (50.3%) received a three-phasic and 1,216 (49.7%) a four-phasic CM injection-protocol (Table 2). The overall number of CM extravasation was 23 out of 2,445 patients (0.9%). The CM extravasation rate in the three-phasic group was 1.4% (17/1,229), whereas in the four-phasic group the extravasation rate was 0.5% (6/1,216), p = 0.034 (Fig. 2). The four-phasic CM injection-protocol resulted in 65% reduction in extravasation rate as compared to the three-phasic CM injection-protocol in coronary CTA (odds ratio (OR): 0.354; CI: 0.139–0.900; p = 0.029). The majority of the patients received an 18 G cannula for CM injection (97.2% of all patients). The use of a 20 G cannula did not differ between the two groups (three-phasic protocol group 34 (3.1%), four-phasic protocol group 38 (3.1%), respectively, p = 0.63).

**Table 2:** Comparison of the extravasation rate, and clinical, vein quality and image acquisition characteristics between the three-phasic contrast media (CM) injection and four-phasic CM injection-protocol groups

	Three-phasic group (n = 1,229)	Four-phasic group (n = 1,216)	p
**Clinical characteristic**			
Age (y)	60.4 ± 12.1	60.8 ± 12.0	0.44
Female, n (%)	529 (44.1)	536 (43.0)	0.63
Height (cm)	171.2 ± 10.0	171.3 ± 10.2	0.88
Weight (kg)	83.8 ± 17.2	84.1 ± 17.2	0.62
BMI (kg/m^2^)	28.5 ± 4.9	28.6 ± 4.7	0.76
Hypertension, n (%)	816 (66.4)	789 (64.9)	0.44
Diabetes, n (%)	202 (16.4)	191 (15.7)	0.66
Dyslipidaemia, n (%)	582 (47.4)	560 (46.1)	0.54
AMI, n (%)	99 (8.1)	86 (7.1)	0.36
PAD, n (%)	120 (10.6)	116 (10.1)	0.73
Stroke or TIA (%)	62 (5.0)	73 (6.0)	0.33
Smoking (%)	497 (40.5)	493 (40.4)	0.97
**Vein quality characteristics**			
Venous access side (right %)	1,103 (89.7)	1,066 (87.7)	0.11
Cubital cannula (%)	1,163 (94.6)	1,130 (92.9)	0.09
**Cannula size**			
18 G, n (%)	1,195 (97.2)	1,178 (96.9)	0.63
20 G, n (%)	34 (3.1)	38 (3.1)	0.63
Cannula size (18 G%)	1,195 (97.2)	1,178 (96.9)	0.63
Successful cannula insertion at first attempt (%)	1,124 (91.5)	1,122 (92.7)	0.77
**Contrast material characteristics**			
Contrast material volume	91.3 ± 7.3	91.0 ± 7.4	0.23
**Injection flow rate**			
5.5 ml/s, n (%)	1,121 (91.2)	1,075 (88.4)	0.02

*AMI* acute myocardial infarction, *BMI* body mass index, *PAD* peripheral artery disease, *TIA* transient ischaemic attack, *DLP* dose-length product

Besides the CM injection-protocol none of the clinical and vein quality characteristics of patients who had extravasation versus patients with no extravasation showed any differences (Table 2). In the four-phasic group a CM injection rate of 5.5 ml/s was administered in 88.4% (1,075/1,216) of the patients, which was lower than the three-phasic protocol group (91.2%, 1,121/1,229), p = 0.02. Even though we found a significant difference between the two groups, this did not influence extravasation rates, since there was no difference in injection rates among patients with extravasation (5.5 ml/s flow rate: 95.7% (22/23)) versus patients who had no extravasation (5.5 ml/s flow rate: 89.8% (2,174/2,422)), p = 0.72.

We assessed the effect of the three- and four-phasic CM injection protocols in subgroups considered prone to developing extravasation. Among females, less extravasation events occurred in the four-phasic group compared to the three-phasic group (5.6% (3/533) vs. 23.2% (12/517), respectively p = 0.02). Similarly, we could detect significantly less extravasation when the four-phasic protocol was administered to patients older than 60 years compared to the three-phasic group (4.0% (3/732) vs. 19.4% (14/720), respectively p = 0.007). Furthermore, we did not experience any extravasation in patients who received a 20 G cannula.

## Discussion

In the present study, we reported a new contrast injection-protocol, which resulted in a 65% reduction of the extravasation rate as compared to the conventionally used three-phasic CM injection-protocol in coronary CTA. The addition of a saline pacer bolus to the three-phasic CM injection-protocol is easy to implement at no additional cost.

We found an overall extravasation rate of 0.9%, which is similar to that in the published literature (0.3–1.3%) [1–5]. Interestingly, from the comprehensive review of Cohan et al. published in 1996 [2] through recent publications, the same range of extravasation rate is reported, which suggests that no effective strategy is available to reduce the risk of CM extravasation [1–5]. To the best of our knowledge our study is the first to describe the four-phasic CM injection-protocol, in which a saline pacer bolus is added to the conventional three-phasic CM protocol to reduce the risk of extravasation.

We detected a statistically significant difference between the three- and four-phasic group regarding contrast injection rates (5.5 ml/s: 91.2% (1,121/1,229) vs. 88.4% (1,075/1,216), respectively, p = 0.02); however, we did not find any difference in injection flow rates among patients with versus without extravasation (5.5 ml/s flow rate: 95.7% (22/23) vs. 89.8% (2,174/2,422), respectively, p = 0.72). In a study by Federle et al., the effect of contrast bolus flow rate was evaluated in 5,106 patients who received CM for CTA examination, and they detected no correlation between extravasation and injection flow rate [3]. The mean CM injection flow rate was 2.8 ml/s (range 1–5 ml/s) and they observed an overall extravasation in 0.9% of the scans. Although the authors used low flow rates, they still experienced the same percentage of extravasation independent of the injection speed as we did in our study. This suggests that instead of the flow rate other characteristics, such as CM viscosity and collapsed vein wall, might play a role in extravasation. We adjusted the CM injection rates according the tube voltage setting. In case of 120 kV we used a higher injection rate (5.5 ml/s) in order to achieve higher intracoronary attenuation. In case of 100 kV we used lower injection rates (4.5 ml/s), due to the increased iodine x-ray absorption at lower tube voltages.

Davenport et al. assessed whether extrinsic warming of low- and high-osmolality CM affects the extravasation rate [15]. They could not detect any beneficial effect of preheating on low-osmolality CM extravasation rates (preheated: 0.30% (32/10,831), non-heated: 0.23% (23/10,064); p = 0.64); however, pre-heating of high-osmolality CM decreased extravasation rate as compared to non-heated (0.27% (5/1,851) vs. 0.87% (18/2,074), respectively; p = 0.05. Similar to these findings, in a prospective study of 4,457 patients iodine concentration and flow rate did not show any association with CM extravasation [4]. In these studies, besides the injection flow rate, the CM injection-protocol was not described in detail, rendering direct comparisons with our study difficult.

Other studies have identified several risk factors of CM extravasation that are unrelated to CM administration protocols and are not modifiable. These risk factors are mainly associated with the fragility of the patients’ vasculature, such as atherosclerosis, diabetes, chemo- or radiotherapy, and autoimmune diseases [2, 12]. Female gender and elderly age (>60 years) were predictors of CM extravasation in a study by Shaquan et al. [11]. Our results suggest that four-phasic protocol reduces extravasation rate independently of these risk factors.

CM extravasation may cause severe complications due to the toxic effects of iodinated CM on the perivascular tissues [2, 5]. Furthermore, it may lead to repeated CTA exams with a consequently higher radiation dose, increased CM load and higher costs. Therefore, the reduction of CM extravasation is of importance. It seems that the beneficial effect of a four-phasic CM injection-protocol is due to the saline pacer bolus, which opens the vein before the high flow-rate CM injection and reduces the risk of vessel wall injury and extravasation, and reduces the risk of vessel wall injury and extravasation. Some state-of-the art power injectors offer ‘keep vein open’ functionality with an intravenous saline drip that is flowing just enough (e.g. 0.25 ml every 30 s) to keep the vein open for a longer time period and prevent coagulation or clot formation at the injection site. Intuitively, this technique might also reduce the risk of extravasation to some extent. However, it is unlikely that the slow drip of saline prevents extravasation as effectively as the saline pacer bolus described in our study, although this needs further investigation. Furthermore, it is important to note that the four-phasic CM injection-protocol is vendor independent and can be programmed with all power injectors.

It is important to note that with the introduction of novel CT technologies, the amount of CM needed to achieve diagnostic quality has markedly decreased. In a recently published study by Kim et al., CM volume usage in coronary CTA performed with 320-row CT could be decreased from 60 to 40 ml with preserved image quality and diagnostic accuracy [16]. In addition, Felmly et al. demonstrated that with the latest generation dual source CT a comprehensive transcatheter aortic valve replacement planning was feasible with reduced CM volumes [17]. In line with these findings, Mangold et al. demonstrated that the use of automated tube voltage selection and CM volume adjustment reduces CM volumes and provides excellent image quality and optimal intravascular attenuation [18]. The effect of novel CT technologies and reduced CM volumes on extravasation rate warrants further investigation.

Our study has some limitations, which should be acknowledged. First, this was a single-centre study, which might limit the generalizability of our results. We used a deterministic method for randomization, which involves open allocation based on odd and even weeks. This might potentially influence recruitment. However, in our study we enrolled all eligible patients, therefore the risk of selection bias is minimized. In addition, we did not perform a power calculation. However, during the 20-month study period we enrolled the maximum number of patients. Furthermore, we defined extravasation based on local symptoms and the inadequate CM enhancement in CTA images. To further objectivize extravasation events a dedicated extravasation monitor system or pressure monitoring would have been beneficial; however, at the time of the study this was not available at our site.

In conclusion, the implementation of four-phasic CM injection-protocol in routine coronary CTA practice is easy and reduces the risk of extravasation at no extra cost. The addition of a saline pacer bolus might be beneficial for all CM injection-protocols in general CT angiography as well; however, this warrants further investigations.

## Abbreviations

AMI: Acute myocardial infarction
CM: Contrast media
CTA: Computer tomography angiography
DLP: Dose-length product
PAD: Peripheral artery disease
TIA: Transient ischaemic attack

## References

[1] Moreno CC, Pinho D, Nelson RC (2013). Lessons learned from 118,970 multidetector computed tomographic intravenous contrast material administrations: impact of catheter dwell time and gauge, catheter location, rate of contrast material administration, and patient age and sex on volume of extravasate. J Comput Assist Tomogr.

[2] Cohan RH, Ellis JH, Garner WL (1996). Extravasation of radiographic contrast material: recognition, prevention, and treatment. Radiology.

[3] Federle MP, Chang PJ, Confer S, Ozgun B (1998). Frequency and effects of extravasation of ionic and nonionic CT contrast media during rapid bolus injection. Radiology.

[4] Wienbeck S, Fischbach R, Kloska SP (2010). Prospective study of access site complications of automated contrast injection with peripheral venous access in MDCT. AJR Am J Roentgenol.

[5] Wang CL, Cohan RH, Ellis JH, Adusumilli S, Dunnick NR (2007). Frequency, management, and outcome of extravasation of nonionic iodinated contrast medium in 69,657 intravenous injections. Radiology.

[6] Shuman WP, Adam JL, Schoenecker SA, Tazioli PR, Moss AA (1986). Use of a power injector during dynamic computed tomography. J Comput Assist Tomogr.

[7] Behrendt FF, Bruners P, Keil S (2009). Impact of different vein catheter sizes for mechanical power injection in CT: in vitro evaluation with use of a circulation phantom. Cardiovasc Intervent Radiol.

[8] Cademartiri F, de Monye C, Pugliese F (2006). High iodine concentration contrast material for noninvasive multislice computed tomography coronary angiography: iopromide 370 versus iomeprol 400. Invest Radiol.

[9] Dykes TM, Bhargavan-Chatfield M, Dyer RB (2015). Intravenous contrast extravasation during CT: a national data registry and practice quality improvement initiative. J Am Coll Radiol.

[10] Wilson BG (2011). Contrast media-induced compartment syndrome. Radiol Technol.

[11] Shaqdan K, Aran S, Thrall J, Abujudeh H (2014). Incidence of contrast medium extravasation for CT and MRI in a large academic medical centre: a report on 502,391 injections. Clin Radiol.

[12] Bellin MF, Jakobsen JA, Tomassin I (2002). Contrast medium extravasation injury: guidelines for prevention and management. Eur Radiol.

[13] Litmanovich D, Zamboni GA, Hauser TH, Lin PJ, Clouse ME, Raptopoulos V (2008). ECG-gated chest CT angiography with 64-MDCT and tri-phasic IV contrast administration regimen in patients with acute non-specific chest pain. Eur Radiol.

[14] Lu JG, Lv B, Chen XB, Tang X, Jiang SL, Dai RP (2010). What is the best contrast injection protocol for 64-row multi-detector cardiac computed tomography?. Eur J Radiol.

[15] Davenport MS, Wang CL, Bashir MR, Neville AM, Paulson EK (2012). Rate of contrast material extravasations and allergic-like reactions: effect of extrinsic warming of low-osmolality iodinated CT contrast material to 37 degrees C. Radiology.

[16] Kim R, Park EA, Lee W, Chung JW (2016). Feasibility of 320-row area detector CT coronary angiography using 40 ml of contrast material: assessment of image quality and diagnostic accuracy. Eur Radiol.

[17] Felmly LM, De Cecco CN, Schoepf UJ (2016). Low contrast medium-volume third-generation dual-source computed tomography angiography for transcatheter aortic valve replacement planning. Eur Radiol.

[18] Mangold S, Wichmann JL, Schoepf UJ (2016). Coronary CT angiography in obese patients using 3(rd) generation dual-source CT: effect of body mass index on image quality. Eur Radiol.

